# Abnormal intrinsic dynamics of dendritic spines in a fragile X syndrome mouse model *in vivo*

**DOI:** 10.1038/srep26651

**Published:** 2016-05-25

**Authors:** Akira Nagaoka, Hiroaki Takehara, Akiko Hayashi-Takagi, Jun Noguchi, Kazuhiko Ishii, Fukutoshi Shirai, Sho Yagishita, Takanori Akagi, Takanori Ichiki, Haruo Kasai

**Affiliations:** 1 Laboratory of Structural Physiology, Center for Disease Biology and Integrative Medicine, Faculty of Medicine, The University of Tokyo, Bunkyo-ku, Tokyo 113-0033, Japan; 2 CREST, Japan Science and Technology Agency, 4-1-8 Honcho, Kawaguchi, Saitama 332-0012, Japan; 3 Department of Bioengineering, School of Engineering, The University of Tokyo, Bunkyo-ku, Tokyo 113-8656, Japan; 4 Laboratory of Medical Neuroscience, Institute for Molecular and Cellular Regulation, Gunma University, Maebachi-city, Gunma 371-8512, Japan; 5 PRESTO, Japan Science and Technology Agency, 4-1-8 Honcho, Kawaguchi, Saitama 332-0012, Japan; 6 Department of Neurosurgery, The University of Tokyo, Bunkyo-ku, Tokyo 113-0033, Japan

## Abstract

Dendritic spine generation and elimination play an important role in learning and memory, the dynamics of which have been examined within the neocortex *in vivo*. Spine turnover has also been detected in the absence of specific learning tasks, and is frequently exaggerated in animal models of autistic spectrum disorder (ASD). The present study aimed to examine whether the baseline rate of spine turnover was activity-dependent. This was achieved using a microfluidic brain interface and open-dura surgery, with the goal of abolishing neuronal Ca^2+^ signaling in the visual cortex of wild-type mice and rodent models of fragile X syndrome (*Fmr1* knockout [KO]). In wild-type and *Fmr1* KO mice, the majority of baseline turnover was found to be activity-independent. Accordingly, the application of matrix metalloproteinase-9 inhibitors selectively restored the abnormal spine dynamics observed in *Fmr1* KO mice, without affecting the intrinsic dynamics of spine turnover in wild-type mice. Such findings indicate that the baseline turnover of dendritic spines is mediated by activity-independent intrinsic dynamics. Furthermore, these results suggest that the targeting of abnormal intrinsic dynamics might pose a novel therapy for ASD.

The majority of excitatory synaptic contacts are formed by small dendritic protrusions in the cerebral cortex, commonly referred to as dendritic spines. The growth and shrinkage of dendritic spines are typically determined by cytosolic Ca^2+^ levels, and they, respectively, underlie the long-term potentiation and depression of synaptic connectivity^1^,^2^,^3^. In addition, the generation and elimination of spines are reported to be induced by tasks involving learning and memory, albeit at a slower rate than processes governing enlargement and shrinkage^4^,^5^,^6^. Spine turnover has been traditionally observed following activity-dependent plasticity induced by cytosolic increases in Ca^2+^ concentration^2^,^6^,^7^, but has also been identified in the absence of specific learning tasks^3^,^8^. Such intrinsic dynamics are reported to occur *in vitro* in a Ca^2+^-independent manner^7^,^9^,^10^. However, to the best of the authors’ knowledge, no study has directly investigated whether the baseline rate of spine turnover reflects non-specific learning under normal rearing conditions, or activity-independent intrinsic dynamics *in vivo*.

Notably, the baseline rate of spine turnover is reported to be augmented in several *in vivo* models of autistic spectrum disorder (ASD)^11^,^12^,^13^. Fragile X syndrome, the most prevalent monogenic form of ASD, is caused by the expansion of CGG repeats upstream of the coding region in the *FMR1* gene, leading to reduction of the fragile X mental retardation protein (FMRP). *Fmr1* knockout (KO) mice present with many of the neural abnormalities observed in patients with fragile X syndrome, including abnormalities in dendritic spine morphology, synaptic plasticity, and learning and memory^14^,^15^,^16^,^17^,^18^. Moreover, spine turnover is similarly increased in *Fmr1* KO mice, as observed in other models of ASD^13^,^19^,^20^. However, no studies have examined whether the increased rate of baseline turnover observed in ASD models reflects activity-dependent plasticity or activity-independent intrinsic dynamics, and therefore the mechanism responsible for increased spine turnover in ASD models remains largely elusive.

With regard to previous *in vivo* neuroimaging techniques, studying the activity-dependent nature of basal spine turnover in the neocortex was difficult using methods such as cranial glass windows or thinned skulls. Because animals are unable to survive when cortical activity is abolished, neuronal Ca^2+^ signaling must be locally silenced in small regions, wherein the time-lapse imaging of dendritic spines can be performed. To resolve this issue, inhibitors of Ca^2+^ signaling were infused locally into the visual cortex via a microfluidic brain interface, and two-photon time-lapse imaging was performed in this region. Ca^2+^ signaling and learning-induced spine turnover were evaluated in wild-type and *Fmr1* KO mice after treatment with Ca^2+^ signal inhibitors. Reports indicate that matrix metalloproteinase 9 (MMP9) KO rescues various abnormalities observed in *Fmr1* KO mice, including structural spine abnormalities^21^. As MMP9 inhibitors have also been linked to changes in spine structure^22^,^23^,^24^, the effect of MMP9 inhibitor administration was also investigated with regard to increased spine turnover in *Fmr1* KO and wild-type mice.

## Results

### Chronic infusion of the adult brain using a microfluidic device

**Figure 1:**
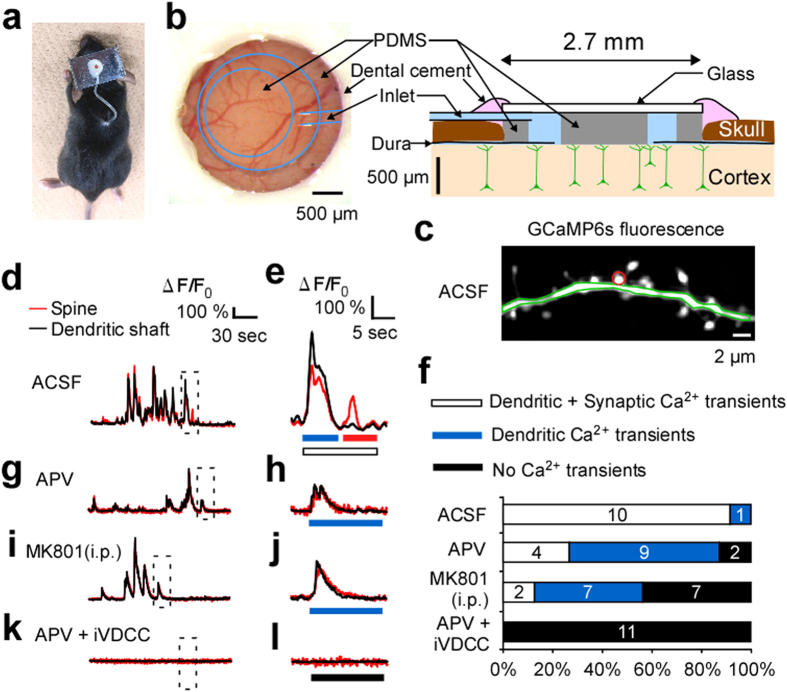
Chronic and local blockade of Ca^2+^ signaling using a brain interface device in the mouse visual cortex. (**a**) A mouse with the interface device connected to an osmotic pump implanted on its back. (**b**) A magnified image of the device and schematic illustration. No drain was used, as the perfusion rate was slow (1.0 μL/h). (**c**) A dendritic branch stained with GCaMP6s and superfused with artificial cerebrospinal fluid (ACSF), where the regions of interests (ROIs) for spine (red) and dendritic shaft (green) are indicated. (**d**,**e**,**g**–**l**) Typical Ca^2+^ transients obtained from mice infused with either ACSF (**d**,**e**; traces from ROIs in (**c**)), APV (**g**,**h**), or APV+ iVDCC (**k**,**l**) and mice who received MK801 intraperitoneally (**i**,**j**). Red traces represent those from spines, and black traces from the dendritic shafts. Dashed areas in (**d**,**g**,**i**,**k**) are magnified in (**e**,**h**,**j**,**l**), respectively. Blue bars in (**e**,**h**,**j**) indicate dendritic Ca^2+^ transients and the red bar in **e** indicates spine Ca^2+^ transients. (**f**) The percentage of the three patterns of Ca^2+^ transients that contain both spine and dendritic transients (open), only dendritic transients (blue), and no transients (black). The patterns were distinguished based on 4-min images of all spines along an imaged dendrite. Data were obtained for the infusion of ACSF (11 dendrites, 10 cells, four mice, 134 spines,), APV (15 dendrites, 11 cells, four mice, 124 spines), or APV+ iVDCC (11 dendrites, nine cells, four mice, 83 spines), and for the injection of MK801 (16 dendrites, 15 cells, five mice, 117 spines). The number on each bar indicates the number of dendrites classified. Fisher exact test, *p* < 0.0001.

The influence of activity on basal spine turnover was investigated using a brain interface device^25^ that enabled the infusion of Ca^2+^ inhibitors into the visual cortex in adult mice (2–6 months old) (Fig. 1a,b). With regard to the surgical method, 20% mannitol was administered to allow the detachment and removal of the dura without directly touching the brain. To maintain a clear cranial window after open-dura surgery, the dural blood vessels were coagulated to prevent bleeding prior to the removal of the dura mater (Supplementary Fig. 1a–c). Two-photon imaging was performed 1 day post-surgery, and chronic infusion was initiated immediately after the first imaging session, to avoid clogging of the inlet of device, using an osmotic pump implanted on the backs of mice (Fig. 1a).

When infusing Alexa594 at the cortical surface, the dilution factor was estimated to be 4% of the original solution in the osmotic pump (Supplementary Fig. 1f–i). The concentration profile of Alexa594 was evaluated across the depth of the cortex, where analysis determined that the concentration did not significantly differ among the cortical layers (Supplementary Fig. 1h,i). Hydrophilic drugs were selected for the infusion experiment, as they diffuse effectively into the brain^26^,^27^.

Under these conditions, no signs of inflammation were detected with regard to microglia or astrocyte activity. *In vivo* imaging of ionized calcium binding adaptor molecule 1 (IBA1)-positive microglia on days 1 and 3 demonstrated relatively limited microglial migration while their processes were dynamic^28^ (Supplementary Fig. 2a). Immunostained slices from the brains of artificial cerebrospinal fluid (ACSF)-infused mice indicated similar levels of IBA1-positive microglia with regard to the surgical and contralateral sides. Comparatively, in positive controls where the cortex was gently depressed during surgery, the number of immunoreactive cells doubled (Supplementary Fig. 2b–d). Slice thickness did not affect the results (IBA1-positive cell ratio of 100 or 50 μm slices, 1.05 ± 0.06 and 0.99 ± 0.05, respectively; glial fibrillary acidic protein (GFAP)-positive cell ratio of 100 or 50 μm slices, 1.03 ± 0.05 and 1.04 ± 0.08, respectively). The number of IBA1-positive microglia did not significantly differ between layers I and II/III (Supplementary Fig. 2e). Similarly, the number of GFAP-positive astrocytes did not significantly differ between the surgical and contralateral sides, and significantly less than in positive controls (Supplementary Fig. 2f–h). The number of GFAP-positive astrocytes was higher in superficial layer I than in layers II/III on both the surgical and contralateral sides (Supplementary Fig. 2i), in line with previous findings^29^.

Dendritic and spine Ca^2+^ transients were imaged in the cortex (Fig. 1c–f) of ACSF infused mice transfected with GCaMP6s^30^ via adeno-associated virus (AAV) transfection. Spine-specific transients were almost abolished when N-methyl-D-aspartate (NMDA) receptors were inhibited, either by infusion of amino-phosphonovaleric acid (APV) at the cortical surface (Fig. 1f–h; 40 μM) or intraperitoneal injection of MK801 (i.e., dizocilpine) (Fig. 1f,i,j; 0.25 mg/kg). In addition, Ca^2+^ signals were eliminated following the administration of APV together with a cocktail of voltage-dependent Ca^2+^ channel inhibitors (Ca_v_1, Ca_v_2.1/2, and Ca_v_2.3; iVDCC) (Fig. 1f,k,l) at concentrations designed to elicit complete blockage (see Methods).

### Intrinsic dynamics of dendritic spines in wild-type mice

**Figure 2:**
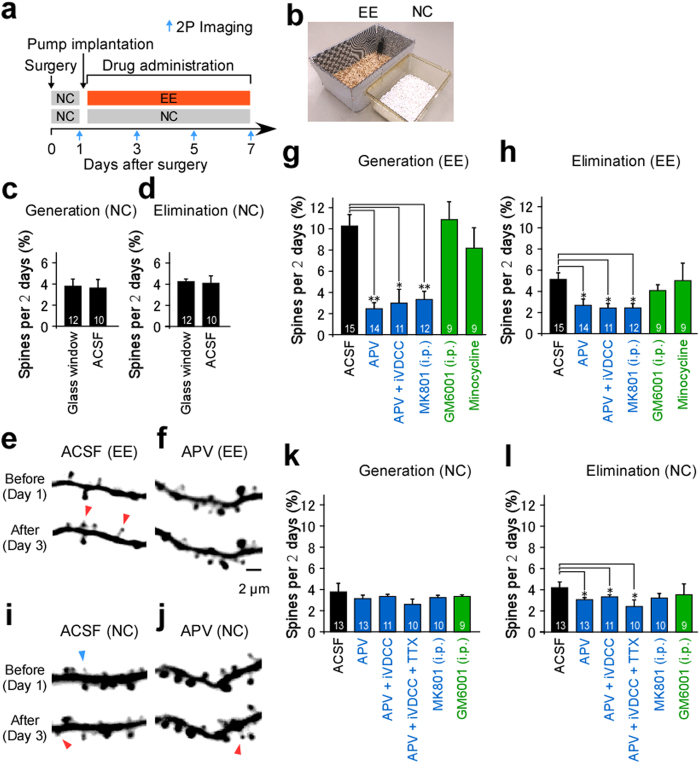
Spine turnover in wild-type mice. (**a**) Time schedules for surgery and imaging. (**b**) Mice cages for visual enrichment (EE) and control (NC) conditions. (**c**,**d**) Spine generation (**c**) and elimination (**d**) rates in mice with glass windows (12 intervals, five cells, five mice, 902 spines) or microfluidic devices for ACSF (10 intervals, four cells, three mice, 1454 spines). (**e**,**f**,**i**,**j**) Examples of dendritic branches from mice reared in EE conditions and superfused with ACSF (**e**) or APV (**f**), and in mice reared in NC and superfused with ACSF (**i**) or APV (**j**). Spines that were eliminated (blue arrowheads) or generated (red arrowheads) are indicated. (**g**,**h**,**k**,**l**) Spine generation (**g**,**k**) and elimination (**h**,**l**) rates for mice reared in EE (**g**,**h**) or NC (**k**,**l**) with the device. Mice received superfusion of ACSF (15 intervals, nine cells, seven mice, 2021 spines from EE; 13 intervals, five cells, five mice, 3154 spines from NC), APV (14 intervals, seven cells, five mice, 1241 spines from EE; 13 intervals, five cells, five mice, 1767 spines from NC), APV+ iVDCC (11 intervals, five cells, five mice, 815 spines from EE; 11 intervals, five cells, five mice, 1442 spines from NC), or APV+ iVDCC+ TTX (10 intervals, five cells, five mice, 1042 spines from NC). Alternatively, mice underwent open-skull surgery with an intraperitoneal injection of MK801 (12 intervals, five cells, five mice, 1412 spines from EE; 10 intervals, five cells, four mice, 1520 spines from NC), GM6001 (10 mg/kg; nine intervals, five cells, three mice, 823 spines from EE; nine intervals, five cells, four mice, 1112 spines from NC), or minocycline treatment (nine intervals, five cells, four mice, 820 spines from EE). Numbers on each bar in (**c**,**d**,**g**,**h**,**k**,**l**) indicate the intervals analyzed. Mann–Whitney test, *p* = 0.9735 in **c** and *p* = 0.7762 in (**d)**. Kruskal–Wallis test, *p* < 0.05 in (**g**,**h**), *p* = 0.9288 in (**k**) and *p* < 0.01 in (**l**). *p < 0.05, **p < 0.01 using a Steel’s test with respect to ACSF (**g**,**h**,**l**).

**Figure 3:**
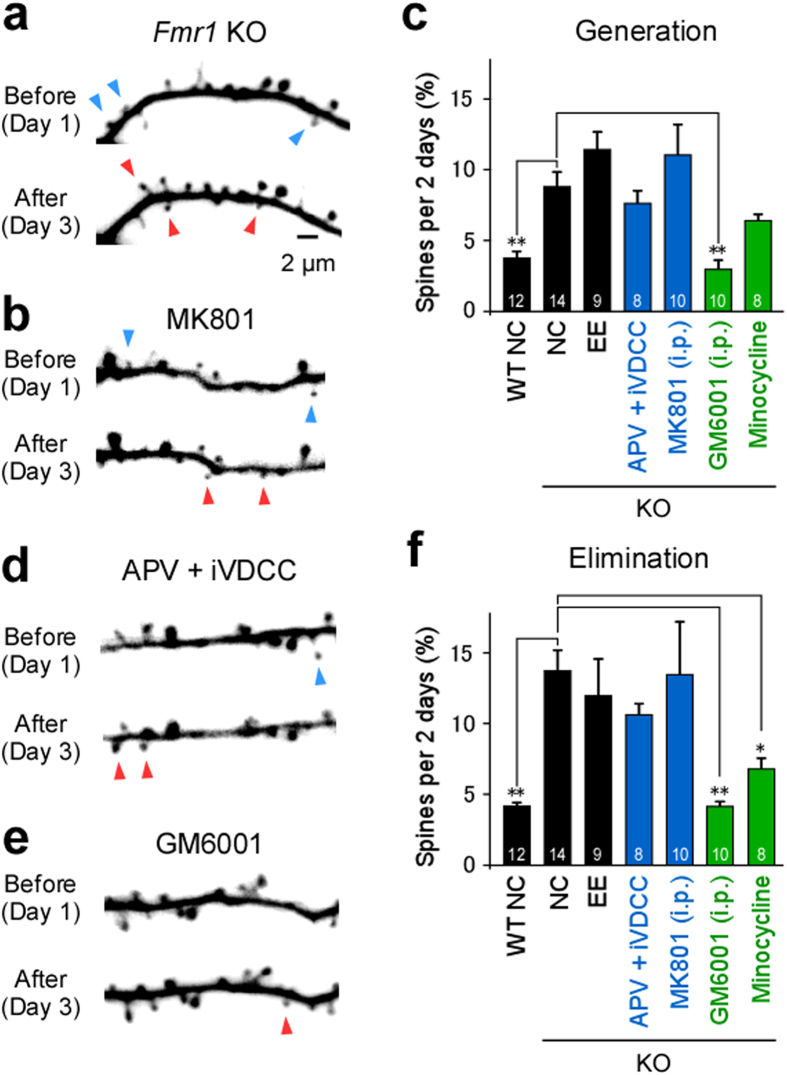
Intrinsic spine turnover in *Fmr1* knockout (KO) mice. (**a**,**b**,**d**,**e**) Examples of dendritic branches imaged on days 1 and 3 in KO mice that underwent open-skull surgery (**a**), with intraperitoneal administration of MK801 (**b**) or GM6001 (**e**), or using the interface device infused with APV+ iVDCC (**d**). Examples of spines eliminated (blue arrowheads) or generated (red arrowheads) over this interval are indicated. (**c**,**f**) Spine generation (**c**) and elimination (**f**) rates over 2-day intervals in wild-type or KO mice with a glass window reared in NC (wild-type: 12 intervals, five cells, five mice, 1526 spines; KO: 14 intervals, six cells, five mice, 2393 spines), KO mice raised in EE conditions (nine intervals, five cells, five mice, 1983 spines), KO mice that received intraperitoneal MK801 (10 intervals, five cells, four mice, 1233 spines), GM6001 (10 intervals, five cells, three mice, 1141 spines), or minocycline through drinking water (eight intervals, five cells, five mice, 1090 spines), in addition to mice that received the interface device with infusion of APV+ iVDCC (eight intervals, five cells, three mice, 996 spines). The numbers on each bar in **c** and **f** indicate the number of intervals analyzed. Spine generation and elimination rates between each imaging session were averaged within each group. Kruskal–Wallis test, *p* < 0.01 in (**c**,**f**). *p < 0.05, **p < 0.01 using a Steel’s test with respect to KO mice that received a glass window.

The first images of the dendritic spines of wild-type (*Thy1-GFP* M-line) mice were obtained immediately prior to infusion, subsequent to which, images were obtained at every 2-day interval (Fig. 2a). In this and ensuing experiments (Figs 2 and 3), all 2-day interval data were combined (Fig. 2a), as no significant differences were identified between data obtained from the first and subsequent imaging intervals (Supplementary Fig. 3). The rates of spine generation and elimination did not significantly differ between mice fitted with conventional glass windows or microfluidic devices (Fig. 2c,d). The efficacy of various inhibitors was assessed with regard to activity-dependent spine remodeling in mice reared in visually enriched environmental (EE) conditions (Fig. 2b,e). Accordingly, the rates of spine generation were relatively high in the EE condition (Fig. 2e,g) compared to the normal condition (NC) (see Fig. 2c,k). In addition, the rate of spine generation was markedly suppressed by infusion of either APV or APV+ iVDCC (Fig. 2f,g), indicating that NMDA receptors play an important role in spine structural plasticity. Similarly, the rates of spine elimination were equally suppressed by the administration of inhibitors (Fig. 2h). The same results were obtained following the intraperitoneal application of MK801 (Fig. 2g,h).

However, spine turnover persisted in the presence of various inhibitors (Fig. 2g,h), which was confirmed in mice reared in under NC (Fig. 2b,i–l). Moreover, the spine generation rate was unaffected by the infusion of APV, APV+ iVDCC, APV+ iVDCC+ TTX, or intraperitoneal injection of MK801 when compared to ACSF superfusion (Fig. 2k). The spine elimination rate was slightly reduced by inhibitor administration (Fig. 2l), indicating that this marginally reflected baseline learning in the NC group.

### Abnormal intrinsic dynamics of dendritic spines in *Fmr1* KO mice

The baseline rate of spine turnover was greater in *Fmr1* KO than in wild-type mice, and was not significantly enhanced by environmental enrichment (Fig. 3). This finding was consistent with previous results^13^,^20^. Increased spine turnover was unaffected by infusion of APV+ iVDCC or intraperitoneal MK801 (Fig. 3b–d,f).

**Figure 4:**
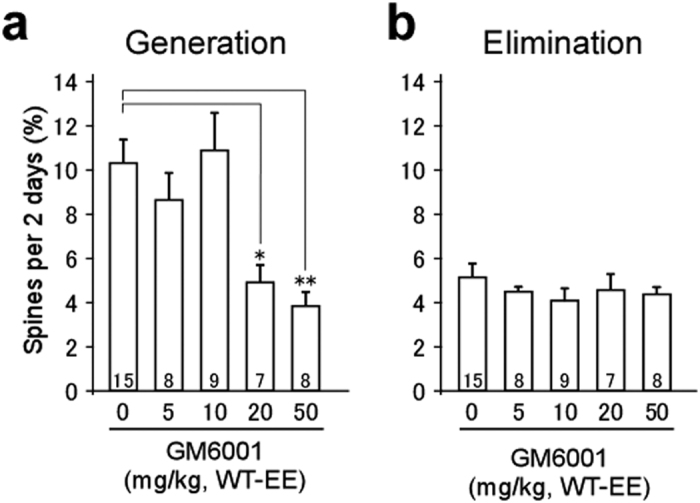
The effect of GM6001 concentration on spine generation and elimination. (**a**,**b**) Spine generation (**a**) and elimination (**b**) rates during 2-day intervals in wild-type mice with a glass window and intraperitoneal (i.p.) injection of GM6001 at 0 mg/kg, 5 mg/kg (eight intervals, five cells, five mice, 598 spines), 10 mg/kg, 20 mg/kg (seven intervals, five cells, five mice, 721 spines), or 50 mg/kg (eight intervals, five cells, three mice, 692 spines). The number on each bar in (**a**,**b**) indicates the number of intervals analyzed. The same data are displayed in Fig. 2g,h with regard to 0 mg/kg and 10 mg/kg. Spine generation and elimination rates between each imaging session were averaged within each group. Kruskal–Wallis test, *p* < 0.01 in (**a**) and *p* = 0.79 in (**b**). *p < 0.05, **p< 0.01 using a Steel’s test with respect to 0 mg/kg.

Next, the effects of GM6001 dose were investigated with regard to intrinsic turnover. In wild-type mice, GM6001, an MMP9 inhibitor, was found to block EE-induced spine generation when administered at high concentrations (20 and 50 mg/kg), but produced no effect at lower concentrations (5 and 10 mg/kg) (Figs 2g,h and 4). This is consistent with the blockade of long-term potentiation identified in previous studies^23^,^24^,^31^. In *Fmr1* KO mice, a low concentration of GM6001 (10 mg/kg) almost completely abrogated excess baseline turnover (Fig. 3c,e,f), while no effect was identified in wild-type mice (Fig. 2k,l). Similar rates of turnover were seen even at high concentrations of GM6001 (20 and 50 mg/kg) in EE mice (Fig. 4). This indicates that normal intrinsic dynamics were unaffected by the application of MMP9 inhibitors in wild-type mice, whereas GM6001 inhibited the abnormal intrinsic dynamics observed in *Fmr1* KO mice. Similar results were obtained with regard to spine generation following the administration of minocycline (Figs 2g,h and 3c,f), which inhibits the synthesis of MMP9^32^.

## Discussion

The present study investigated the activity-independent intrinsic dynamics of dendritic spine turnover via the implantation of a neural interface device to enable the local infusion of Ca^2+^ antagonists into the visual cortex of adult wild-type and *Fmr1* KO mice. While the dura was removed, and infusions were delivered to the cortical surface via an osmotic pump, no significant activation of glial cells was detected. The results indicate that the evaluation of inflammation was sufficiently sensitive in the present study, and that open-dura microfluidic surgery did not cause excessive neural damage nor induce a significant immune response in the week post-surgery. In addition, the spine turnover rate in the neural interface group was similar to that of groups receiving conventional glass windows, or thinned-skull surgery in previous studies^33^. This confirms that neither device implantation nor local infusion affected the rate of spine turnover. After one week, the inlet of the device often clogged, and prevented longer-term analysis, which should be improved in the future studies.

Inhibitors were applied to target a variety of factors, including NMDA receptors, Na^+^ channels, and P-type (Ca_v_2.1), N- (Ca_v_2.2), R- (Ca_v_2.3), and L-type (Ca_v_1) Ca^2+^ channels in the postsynaptic dendrites. Inhibitor administration was designed to abolish the evoked release of neurotransmitters in the presynaptic terminal. The only remaining sources of Ca^2+^ signaling were miniature synaptic transmissions and T-type (Ca_v_3) Ca^2+^ channels. No increase in cytosolic Ca^2+^ concentration was detected in dendrites and spines treated with the inhibitor cocktail. This was monitored via the transfection of the Ca^2+^ indicator proteins GCaMP6s, which feature the highest Ca^2+^ sensitivity reported to date^34^. We also confirmed that the application of inhibitors abrogated the EE-induced increase in spine turnover in wild-type mice. These results clearly demonstrate that baseline turnover occurs independently of variations in cytosolic Ca^2+^. As baseline spine generation persisted in the complete absence of neuronal Ca^2+^ signaling, even when both Na^+^ and Ca^2+^ channels were simultaneously blocked, this suggests that the administration of inhibitors successfully blocked activity-dependent plasticity, and that baseline turnover was predominantly governed by Ca^2+^-independent intrinsic structural dynamics. Previous studies have indicated that such intrinsic dynamics might be observed in other cortical regions. Indeed, fractional spine turnover has been detected in the somatosensory and motor cortices in the presence of NMDA receptor inhibitors^6^,^35^. The present study confirmed that the injection of intraperitoneal MK801 sufficiently blocked NMDA receptors, and reduced the increase in spine generation detected in wild-type mice reared in EE conditions. Because these findings were similar to those obtained using the microfluidic interface device, this suggests that suppression was not caused by the adverse effects of local infusion. Intraperitoneal MK801 infusion could therefore be used to characterize intrinsic spine dynamics without the need for open-dura surgery in future studies.

It is likely that such dynamics are produced by intrinsic fluctuations in spine volume and consequent turnover, as these phenomena are strongly related *in vitro*^7^. As spine functions are independently modifiable, they therefore behave as physical correlates of memory^36^. However, unlike memories in a manmade computer, dendritic spines are living structures and are inevitably unstable. Fluctuations in spine dynamics might arise due to actin treadmilling^37^,^38^, dynamic microtubule activity^39^, turnover of postsynaptic-density molecules^40^,^41^, dynamic maintenance of membrane structures^3^,^8^, or mitochondrial motility^42^. Instability of the extracellular matrix, which regulates spine stability, might underlie the fluctuations in spine dynamics. However, the mechanisms underlying MMP9 involvement might not significantly contribute to intrinsic fluctuations in wild-type mice. In addition, despite the complete blockade of action potentials, constitutive release of neurotransmitters^43^, hormones^44^, neurotrophins^45^ and cytokines might modulate spine dynamics.

Spines are surrounded by pulsating blood vessels and glial cells, which are motile and interact dynamically with spines^28^,^46^. It is remarkable that spine stability can be maintained throughout the lifespan of a mouse under these circumstances^47^. Intrinsic fluctuations in spine dynamics might underlie memory decay^7^, or the maintenance of cortical networks, which remain to be elucidated^7^. Importantly, the intrinsic dynamics of spines observed in the adult neocortex *in vivo* were activity-independent in the present study, unlike homeostatic synaptic plasticity^48^,^49^. Moreover, intrinsic dynamics were detected in individual synapses, whereas homeostatic plasticity is a global regulation process of glutamate sensitivity^48^,^50^.

In particular, the present study provides evidence for abnormal intrinsic spine dynamics in *Fmr1* KO mice. These abnormal spine dynamics are likely a result of MMP9 overexpression, as abnormalities associated with the *Fmr1* KO phenotype were selectively blocked by MMP9 inhibitors. Such abnormalities might account for the learning disabilities and social impairment reported in individuals diagnosed with ASD^20^,^51^, as recent studies indicate that behavioral rescue of *Fmr1* KO mice was possible following *MMP9* KO^21^, and minocycline treatment^14^. The learning deficits associated with ASD might also be related to abnormalities in activity-dependent plasticity^52^,^53^,^54^,^55^. Since MMP9 also impairs activity-dependent plasticity, it is necessary to develop new drugs to selectively prevent the abnormal intrinsic dynamics for clinical application to ASD.

Therefore, the present study identified intrinsic spine dynamics *in vivo* that are fundamental to the understanding of neural function and subsequent disorders. Although the exact mechanisms underlying spine turnover in ASD require further study, the present results provide novel insight with regard to the connection between increased baseline spine turnover and associated learning deficits.

## Methods

### Subjects

A colony of homozygous transgenic mice expressing green fluorescent protein (GFP) under the control of the *Thy1* promoter (*Thy1-GFP* M-line mice) was generated for the present study^56^. B6.129P2-*Fmr1*^*tm1Cgr*^/J (Jackson Lab) KO female mice (*Fmr1*^+/−^) were crossed with males homozygous for *Thy-1 GFP* to generate *GFP-FMR1*^*−/y*^ mice. Transgenic mice expressing GFP under the control of the *Iba1* promoter (*Iba1-GFP* mice)^57^ were used for the *in vivo* imaging of microglia (Supplementary Fig. 2a). Under NC, mice were reared in a 20- × 30- × 15-cm transparent plastic cage with shredded paper as bedding. For the EE condition, mice were reared in a larger cage (25 × 41 × 18 cm) with patterned wallpaper and a sawdust bedding (Fig. 2b). Imaging experiments were performed 1 day post-surgery in 2- to 6-month-old mice. After imaging, mice were returned to their respective cages. Intraperitoneal injections of MK801 were performed twice daily (0.25 mg/kg dissolved in saline)^6^, while injections of GM6001 (5–50 mg/kg dissolved in 1–10% DMSO in saline) were performed once daily. Minocycline was dissolved in drinking water (~50 mg/kg/day). All procedures were approved by the Animal Experiment Committee of the University of Tokyo. Procedures were carried out in accordance with the University of Tokyo Animal Care and Use Guidelines.

### AAV injection

For Ca^2+^ imaging experiments, an AAV vector was used, engineered to express the genetically encoded calcium indicators GCaMP6s in a Cre-dependent manner (virus 1, AAV1.Syn.Flex.GCaMP6S.WPRE with a titer of 2.98E + 13 [GC/ml]) and a Cre driver (virus 2, AAV1.CamKII0.4.Cre.SV40 with a titer of 1.84E + 13 [GC/ml], after dilution with ACSF 10,000 times. These vectors were purchased from U Penn Vector Core (PA, USA). Six-week-old male mice were anesthetized with ketamine (60 mg/kg) and xylazine (10 mg/kg). After shaving the head and applying xylocaine jelly 2% (AstraZeneca, London, UK) to the exposed skin, a round hole with a diameter of 0.5 mm was created using a drill (Coordinates 3 mm posterior and 2.5 mm lateral to bregma). AAV Injection was performed 250 μm from the dural surface using a glass needle (outer diameter, 40 μm). The viral solution was injected at a speed of 50 nL/min for a total volume of 100 or 200 nL (mix of virus 1 and virus 2 at a 9:1 ratio) using a syringe pump (Legato 130, KD Scientific, MA, USA). The needle remained in place for 3 min before the needle was removed, and the hole was plugged with cyanoacrylate (Aron-alpha, Toagosei Company, Tokyo, Japan). Ca^2+^ imaging was performed 3 weeks after virus injection.

### Microfluidic device

The microfluidic interface device was designed as previously described^25^ with the following modification. A perfluorocarbon tube with an outer diameter of 200 μm was connected to the poly (dimethylsiloxane) (PDMS) ring as an inlet (Fig. 1b). No outlet was placed, because the infusion rate (1.0 μL/h) was relatively slow compared to the generation of cerebrospinal fluid (18 μL/h)^58^. Beneath the glass cover (150 μm), a PDMS disk (diameter, 1.2 mm; thickness, ~400 μm) was inserted to prevent brain pulsation, in addition to a ring-shaped layer of PDMS with a 2.0-mm inner and 2.7-mm outer diameter (Fig. 1b). These layers were bonded together by heating at 235 °C for 150 min.

### Cranial open-dura surgery for chronic drug application

Mice were anesthetized with isoflurane (4.5% for induction; 1.0–1.5% for maintenance), delivered via a face mask using an anesthetic regulator (Narcobit-E, Natsume Seisakusho, Tokyo, Japan). The level of anesthesia was assessed by monitoring the tail-pinch reflex. Administration of 20% mannitol (30 μL/g body weight) was achieved intraperitoneally, while ketoprofen, (2 μL/g body weight) was administered subcutaneously. Mannitol was applied to shrink the brain and improve separation between the cortex and dura prior to the removal of the dura with a pair of forceps during open-dura surgery. Mice were maintained at 37 °C on a heating pad and the head was stabilized in a stereotaxic frame. Ointment (Tarivid, Santen Pharmaceutical, Osaka, Japan) was applied to the eyes. Once the tail reflex disappeared, the scalp was shaved, washed with ethanol, and removed using a pair of scissors, after which the periosteum was gently removed. A head plate with a 5-mm diameter hole was fixed to the skull using dental cement (Fuji Lute BC, GC Corp., Tokyo, Japan). A small craniotomy (2.7-mm diameter) was performed over the left visual cortex based on stereotaxic coordinates^59^, which were confirmed by intrinsic signal imaging. The craniotomy was achieved using a trephine drill (224RF-027, Meisinger, Neuss, Germany) fixed to a stereotaxic instrument. The skull covering the visual cortex was gently removed, and the dura mater was detached from the brain using fine forceps. To avoid bleeding after the surgery, dural vessels in the imaging area were coagulated using heated forceps (Supplementary Fig. 1a,b). The dura mater underneath the device (Supplementary Fig. 1c) was then partially removed with a pair of forceps.

The device was filled with ACSF (125 mM NaCl, 2.5 mM KCl, 1 mM MgCl_2_, 2 mM CaCl_2_, 1.25 mM NaH_2_PO_4_, 26 mM NaHCO_3_, and 20 mM D-Glucose), and the inlet was plugged with silver wire. The device was positioned within the cranial window and sealed in place with dental cement and dental acrylic (ADFA, Shofu, Kyoto, Japan). The device was protected with a metal cover when the mice were returned to their cages (Supplementary Fig. 1e). Animals were housed separately after surgery. The infusion solutions were sterilized using a 0.22-μm syringe filter. Surgical instruments were pre-sterilized using a glass bead sterilizer (Steri 250, Simon Keller, Burgdorf, Switzerland).

Chronic infusion was performed using an osmotic pump (infusion rate, 1 μL/h; Alzet mini-osmotic pump, model 2001, Alza, CA, USA). Osmotic pumps were implanted subcutaneously onto the backs of mice, and connected to the infusion device at the end of the first imaging session on day 1. Each infusion consisted of one of the following solutions: ACSF, APV, APV+ iVDCC, or APV+ iVDCC+ TTX. In this context, APV refers to amino-5-phosphonopentanoate (1 mM), an NMDA antagonist. iVDCC includes calcicludine (5 μM)^60^ a Ca_v_1 channel blocker, ω-conotoxin MVIIC (25 μM)^61^ a selective Ca_v_2.1/2 blocker, and SNX-482 (7.5 μM)^62^ a Ca_v_2.3 blocker; whilst TTX refers to the Na^+^ channel blocker tetrodotoxin (50 μM). The final inhibitor concentrations at the surface of the brain were predicted to be 4% of that in the osmotic pump, and should therefore completely block their respective ion channels. For conventional open-skull surgery with a glass window, the dura was kept intact and mannitol was not used during surgery. Circular glass (diameter of 2.7 mm, Matsunami Glass, Osaka, Japan) was fixed with dental cement to the cranial window.

### Labeling of microglia and astrocytes

Inflammation was monitored via glial cell imaging. Mice were anesthetized with isoflurane, and perfused with PBS followed by 4% paraformaldehyde (Wako, Osaka, Japan). The brains were removed, fixed overnight in 4% paraformaldehyde at 4 °C, and sliced into 50- or 100-μm-thick coronal sections using a vibratome (VT1000S, Leica, Nussloch, Germany). Slices were then pre-incubated in blocking buffer (2% horse serum, 0.01% Tween 20, 0.1% NaN_3_ in PBS) for 30 min. Microglia and astrocytes were immunolabeled overnight using rabbit anti-Iba-1 antibody (1:1,000 in blocking buffer, cat. #019-19741, Wako) or rabbit anti-GFAP (1:250 in blocking buffer, cat. #ab68428, Abcam, Cambridge, UK), respectively. The samples were washed five times with PBS for 5 min, then incubated with the secondary antibody (1:200 in PBS, Alexa594-labeled goat anti-rabbit IgG, Molecular Probes, OR, USA) for 30 min, then washed another five times with PBS. Slices were mounted onto glass slides and imaged with a two-photon microscope at 830 nm. The numbers of glial cells were calculated between the subpial zone and a region 200 μm from the pial surface, after which the values were compared with those on the contralateral side. With regard to the brain interface device, either microglial or astrocyte staining was performed after the imaging sessions, and the data were discarded if signs of inflammation were found (>100 GFAP-positive cells/mm^2^, except for positive controls, Supplementary Fig. 2h). To validate the sensitivity of immunostaining, the brain was intentionally depressed (~200 μm, ~2 s) using a pair of forceps in several of the mice as a positive control.

### Two-photon imaging *in vivo*

Two-photon imaging was performed using upright microscopes (BX61WI, Olympus, Tokyo, Japan; or LSM710NLO, Zeiss, Jena, Germany) equipped with Ti-sapphire lasers (Mai-Tai-DS-HP, SpectraPhysics, CA, USA) set at 950 nm with either a 60× (LUMPlanFI/IR, 0.9 NA, Olympus) or 25× (XLPLN25XWMP2, 1.05 NA, Olympus) water immersion lens. Average excitation power was maintained at <40 mW under the objective. This power was selected to avoid saturating the fluorescence of dendrites. The fluorescence intensities of dendritic shafts were similar between regions of interests (ROI) and across animals. Mice were anesthetized with a 0.8–1.2% isoflurane-oxygen mixture (Univentor 400 anesthesia unit, Univentor, Zejtun, Malta), and body temperature was maintained at 37 °C using a heating pad. For spine Ca^2+^ imaging, anesthesia was maintained with 0.4–0.8% isoflurane. The head was restrained during image acquisition (Supplementary Fig. 1d). Images were obtained from layer V pyramidal cells with apical dendrites in layer I. Dendrites on which spines were well separated from another were selected for analysis. Infusions were administered immediately after the first imaging session on day 1 (Fig. 2a).

Image processing and analysis were performed using ImageJ. For spine Ca^2+^ imaging, images were acquired at 10–15 frames/s and the backgrounds were subtracted. Oval ROIs were placed over each spine, while polygonal ROIs were placed over the dendritic shaft (Fig. 1c). ΔF/F_0_ was calculated for each ROI from each frame as (F − F_0_)/F_0_, where F_0_ was the mode of fluorescence signal during the 4-min imaging session. Calcium signals that crossed three standard deviations for more than two consecutive frames were defined as dendritic shaft responses. Active dendritic shafts were defined as those producing one or more responses during the 4-min imaging session. Dendritic Ca^2+^ transients invaded the spines in most instances (Fig. 1d–j). To identify spine-specific calcium responses, the dendritic shaft traces were scaled as much as possible to fit with the spine signals. If calcium transients in the spines exceeded dendritic transients on more than three occasions, such spines were classified as active spines. All spines on the selected dendrites were analyzed. Such measurements were performed on one to four dendrites per cell. If any active spines were identified within these images, the dendrite was categorized as “dendritic + synaptic Ca^2+^ transients” (Fig. 1f). If no active spines were identified, but the dendrite was active, the dendrite was categorized as “dendritic Ca^2+^ transients” (Fig. 1g–j). If neither active spines nor shafts were identified, the dendrite was categorized as “no Ca^2+^ transients” (Fig. 1k,l).

For dendrite structural imaging, three-dimensional reconstructions of dendritic morphology were generated from a stack of 19–71 two-dimensional images, each separated by 0.5 μm. Spine turnover was assessed on every 2-day interval post-surgery (Fig. 2a). Spines were identified as protrusions from dendrites with an apparent head structure (head diameter/neck diameter >1.2 and head fluorescence/neck fluorescence >1.2). Spines emanating from the dendrite perpendicular to the imaging plane were counted only when the head was clearly visible in a section. Filopodial protrusions without a head structure were excluded from analysis. Spines were considered the same between sessions if their positions remained the same distance from adjacent landmarks. The rates of dendritic spine generation and elimination were defined as the percentage of spines that appeared and disappeared, respectively, between two successive imaging sessions with an interval of 2 days. Dendritic shaft lengths were analyzed using ImageJ Simple Neurite Tracer plugin. The average length of the analyzed dendritic shafts was 488 ± 60 μm (*N*_*cell*_ = 126). Data were discarded if any signs of damage were detected, included bleeding, a dim window, swelling/blebbing, or a feeble fluorescence level compared to the previous imaging session. Spine turnover rates at 2–4 months and 4–6 months old did not significantly differ, except for a slight reduction in elimination rate with regard to APV infused mice raised in NC, and a reduction in generation for KO mice raised in EE conditions (Supplementary Fig. 4a–h).

### Statistical analyses

Spine generation and elimination rates were assessed at 2-day intervals, and averaged within each group, except for Supplementary Fig. 3, wherein spine generation and elimination rates were compared between the initial 2-day imaging interval and subsequent intervals. All data are presented as mean ± SEM, and were analyzed using a Fisher’s exact test (Fig. 1f), Kruskal–Wallis test (Figs 2, 3, 4), or underwent further analysis using a Steel’s test. A Mann–Whitney test was used to analyze the data presented in Supplementary Figs 2–4. No statistical methods were used to predetermine sample sizes, but the sample sizes used were similar to those reported in previous publications. Randomization and blinding were not used in this study.

## Additional Information

**How to cite this article**: Nagaoka, A. *et al.* Abnormal intrinsic dynamics of dendritic spines in a fragile X syndrome mouse model *in vivo*. *Sci. Rep.* **6**, 26651; doi: 10.1038/srep26651 (2016).

## Supplementary Material

Supplementary Information

## References

[b1] Matsuzaki M., Honkura N., Ellis-Davies G. C. & Kasai H. Structural basis of long-term potentiation in single dendritic spines. Nature 429, 761–766, doi: 10.1038/nature02617 (2004). 15190253 PMC4158816

[b2] Hayama T. *et al.* GABA promotes the competitive selection of dendritic spines by controlling local Ca^2+^ signaling. Nat Neurosci 16, 1409–1416, doi: 10.1038/nn.3496 (2013). 23974706 PMC4135703

[b3] Holtmaat A. & Svoboda K. Experience-dependent structural synaptic plasticity in the mammalian brain. Nat Rev Neurosci 10, 647–658, doi: 10.1038/nrn2699 (2009). 19693029

[b4] Hofer S. B., Mrsic-Flogel T. D., Bonhoeffer T. & Hubener M. Experience leaves a lasting structural trace in cortical circuits. Nature 457, 313–317, doi: 10.1038/nature07487 (2009). 19005470 PMC6485433

[b5] Trachtenberg J. T. *et al.* Long-term *in vivo* imaging of experience-dependent synaptic plasticity in adult cortex. Nature 420, 788–794 (2002). 12490942 10.1038/nature01273

[b6] Zuo Y., Yang G., Kwon E. & Gan W. B. Long-term sensory deprivation prevents dendritic spine loss in primary somatosensory cortex. Nature 436, 261–265, doi: 10.1038/nature03715 (2005). 16015331

[b7] Yasumatsu N., Matsuzaki M., Miyazaki T., Noguchi J. & Kasai H. Principles of long-term dynamics of dendritic spines. J Neurosci 28, 13592–13608, doi: 10.1523/JNEUROSCI.0603-08.2008 (2008). 19074033 PMC2706274

[b8] Kasai H. *et al.* Learning rules and persistence of dendritic spines. Eur J Neurosci 32, 241–249, doi: 10.1111/j.1460-9568.2010.07344.x (2010). 20646057

[b9] Minerbi A. *et al.* Long-term relationships between synaptic tenacity, synaptic remodeling, and network activity. PLoS Biol 7, e1000136 (2009). 19554080 10.1371/journal.pbio.1000136 PMC2693930

[b10] Dunaevsky A., Tashiro A., Majewska A., Mason C. & Yuste R. Developmental regulation of spine motility in the mammalian central nervous system. Proc Natl Acad Sci USA 96, 13438–13443 (1999). 10557339 10.1073/pnas.96.23.13438 PMC23966

[b11] Isshiki M. *et al.* Enhanced synapse remodelling as a common phenotype in mouse models of autism. Nat Comm 5, 4742, doi: 10.1038/ncomms5742 (2014). 25144834

[b12] Jiang M. *et al.* Dendritic arborization and spine dynamics are abnormal in the mouse model of MECP2 duplication syndrome. J Neurosci 33, 19518–19533, doi: 10.1523/JNEUROSCI.1745-13.2013 (2013). 24336718 PMC3858623

[b13] Pan F., Aldridge G. M., Greenough W. T. & Gan W. B. Dendritic spine instability and insensitivity to modulation by sensory experience in a mouse model of fragile X syndrome. Proc Natl Acad Sci USA 107, 17768–17773, doi: 10.1073/pnas.1012496107 (2010). 20861447 PMC2955121

[b14] Bilousova T. V. *et al.* Minocycline promotes dendritic spine maturation and improves behavioural performance in the fragile X mouse model. J Med Gen 46, 94–102, doi: 10.1136/jmg.2008.061796 (2009). 18835858

[b15] Comery T. A. *et al.* Abnormal dendritic spines in fragile X knockout mice: maturation and pruning deficits. Proc Natl Acad Sci USA 94, 5401–5404 (1997). 9144249 10.1073/pnas.94.10.5401 PMC24690

[b16] Dutch-Belgian Fragile X Consortium. *Fmr1* knockout mice: a model to study fragile X mental retardation. Cell 78, 23–33 (1994). 8033209

[b17] Sidorov M. S., Auerbach B. D. & Bear M. F. Fragile X mental retardation protein and synaptic plasticity. Mol Brain 6, 15, doi: 10.1186/1756-6606-6-15 (2013). 23566911 PMC3636002

[b18] Zhao M. G. *et al.* Deficits in trace fear memory and long-term potentiation in a mouse model for fragile X syndrome. J Neurosci 25, 7385–7392, doi: 10.1523/JNEUROSCI.1520-05.2005 (2005). 16093389 PMC6725289

[b19] Cruz-Martin A., Crespo M. & Portera-Cailliau C. Delayed stabilization of dendritic spines in fragile X mice. J Neurosci 30, 7793–7803, doi: 10.1523/JNEUROSCI.0577-10.2010 (2010). 20534828 PMC2903441

[b20] Padmashri R., Reiner B. C., Suresh A., Spartz E. & Dunaevsky A. Altered structural and functional synaptic plasticity with motor skill learning in a mouse model of fragile x syndrome. J Neurosci 33, 19715–19723, doi: 10.1523/JNEUROSCI.2514-13.2013 (2013). 24336735 PMC3858638

[b21] Sidhu H., Dansie L. E., Hickmott P. W., Ethell D. W. & Ethell I. M. Genetic removal of matrix metalloproteinase 9 rescues the symptoms of fragile X syndrome in a mouse model. J Neurosci 34, 9867–9879, doi: 10.1523/JNEUROSCI.1162-14.2014 (2014). 25057190 PMC4107404

[b22] Michaluk P. *et al.* Influence of matrix metalloproteinase MMP-9 on dendritic spine morphology. J Cell Sci 124, 3369–3380, doi: 10.1242/jcs.090852 (2011). 21896646

[b23] Szepesi Z., Bijata M., Ruszczycki B., Kaczmarek L. & Wlodarczyk J. Matrix metalloproteinases regulate the formation of dendritic spine head protrusions during chemically induced long-term potentiation. PLoS One 8, e63314, doi: 10.1371/journal.pone.0063314 (2013). 23696812 PMC3656002

[b24] Wang X. B. *et al.* Extracellular proteolysis by matrix metalloproteinase-9 drives dendritic spine enlargement and long-term potentiation coordinately. Proc Natl Acad Sci USA 105, 19520–19525, doi: 10.1073/pnas.0807248105 (2008). 19047646 PMC2614793

[b25] Takehara H. *et al.* Lab-on-a-brain: implantable micro-optical fluidic devices for neural cell analysis *in vivo*. Sci Rep 4, 6721, doi: 10.1038/srep06721 (2014). 25335545 PMC4205880

[b26] Noguchi J. *et al.* *In vivo* two-photon uncaging of glutamate revealing the structure-function relationships of dendritic spines in the neocortex of adult mice. J Physiol 589, 2447–2457, doi: 10.1113/jphysiol.2011.207100 (2011). 21486811 PMC3115818

[b27] Syková E. & Nicholson C. Diffusion in brain extracellular space. Physiol Rev 88, 1277–1340, doi: 10.1152/physrev.00027.2007 (2008). 18923183 PMC2785730

[b28] Nimmerjahn A., Kirchhoff F. & Helmchen F. Resting microglial cells are highly dynamic surveillants of brain parenchyma *in vivo*. Science 308, 1314–1318, doi: 10.1126/science.1110647 (2005). 15831717

[b29] Holtmaat A. *et al.* Long-term, high-resolution imaging in the mouse neocortex through a chronic cranial window. Nat Protoc 4, 1128–1144, doi: 10.1038/nprot.2009.89 (2009). 19617885 PMC3072839

[b30] Chen T. W. *et al.* Ultrasensitive fluorescent proteins for imaging neuronal activity. Nature 499, 295–300, doi: 10.1038/nature12354 (2013). 23868258 PMC3777791

[b31] Wojtowicz T. & Mozrzymas J. W. Matrix metalloprotease activity shapes the magnitude of EPSPs and spike plasticity within the hippocampal CA3 network. Hippocampus 24, 135–153, doi: 10.1002/hipo.22205 (2014). 24115249

[b32] Dziembowska M. *et al.* High MMP-9 activity levels in fragile X syndrome are lowered by minocycline. Am J Med Gen Part A 161, 1897–1903, doi: 10.1002/ajmg.a.36023 (2013). 23824974

[b33] Yang G., Pan F. & Gan W. B. Stably maintained dendritic spines are associated with lifelong memories. Nature 462, 920–924, doi: 10.1038/nature08577 (2009). 19946265 PMC4724802

[b34] Inoue M. *et al.* Rational design of a high-affinity, fast, red calcium indicator R-CaMP2. Nat Methods 12, 64–70, doi: 10.1038/nmeth.3185 (2014). 25419959

[b35] Yang G. *et al.* Sleep promotes branch-specific formation of dendritic spines after learning. Science 344, 1173–1178, doi: 10.1126/science.1249098 (2014). 24904169 PMC4447313

[b36] Hayashi-Takagi A. *et al.* Labelling and optical erasure of synaptic memory traces in the motor cortex. Nature 525, 333–338, doi: 10.1038/nature15257 (2015). 26352471 PMC4634641

[b37] Honkura N., Matsuzaki M., Noguchi J., Ellis-Davies G. C. & Kasai H. The subspine organization of actin fibers regulates the structure and plasticity of dendritic spines. Neuron 57, 719–729, doi: 10.1016/j.neuron.2008.01.013 (2008). 18341992

[b38] Urban N. T., Willig K. I., Hell S. W. & Nagerl U. V. STED nanoscopy of actin dynamics in synapses deep inside living brain slices. Biophys J 101, 1277–1284, doi: 10.1016/j.bpj.2011.07.027 (2011). 21889466 PMC3164186

[b39] Hu X. *et al.* BDNF-induced increase of PSD-95 in dendritic spines requires dynamic microtubule invasions. J Neurosci 31, 15597–15603, doi: 10.1523/JNEUROSCI.2445-11.2011 (2011). 22031905 PMC3224154

[b40] Okabe S., Kim H. D., Miwa A., Kuriu T. & Okado H. Continual remodeling of postsynaptic density and its regulation by synaptic activity. Nat Neurosci 2, 804–811 (1999). 10461219 10.1038/12175

[b41] Gray N. W., Weimer R. M., Bureau I. & Svoboda K. Rapid redistribution of synaptic PSD-95 in the neocortex *in vivo*. PLoS Biol 4, e370 (2006). 17090216 10.1371/journal.pbio.0040370 PMC1634879

[b42] Obashi K. & Okabe S. Regulation of mitochondrial dynamics and distribution by synapse position and neuronal activity in the axon. Eur J Neurosci 38, 2350–2363, doi: 10.1111/ejn.12263 (2013). 23725294

[b43] Kasai H., Takahashi N. & Tokumaru H. Distinct initial SNARE configurations underlying the diversity of exocytosis. Physiol Rev 92, 1915–1964, doi: 10.1152/physrev.00007.2012 (2012). 23073634

[b44] Liston C. & Gan W. B. Glucocorticoids are critical regulators of dendritic spine development and plasticity *in vivo*. Proc Natl Acad Sci USA 108, 16074–16079, doi: 10.1073/pnas.1110444108 (2011). 21911374 PMC3179117

[b45] Shimada A., Mason C. A. & Morrison M. E. TrkB signaling modulates spine density and morphology independent of dendrite structure in cultured neonatal Purkinje cells. J Neurosci 18, 8559–8570 (1998). 9786964 10.1523/JNEUROSCI.18-21-08559.1998 PMC6793520

[b46] Perez-Alvarez A., Navarrete M., Covelo A., Martin E. D. & Araque A. Structural and functional plasticity of astrocyte processes and dendritic spine interactions. J Neurosci 34, 12738–12744, doi: 10.1523/JNEUROSCI.2401-14.2014 (2014). 25232111 PMC6705321

[b47] Grutzendler J., Kasthuri N. & Gan W. Long-term dendritic spine stability in the adult cortex. Nature 420, 812–816 (2002). 12490949 10.1038/nature01276

[b48] Turrigiano G. G., Leslie K. R., Desai N. S., Rutherford L. C. & Nelson S. B. Activity-dependent scaling of quantal amplitude in neocortical neurons. Nature 391, 892–896 (1998). 9495341 10.1038/36103

[b49] Vitureira N. & Goda Y. Cell biology in neuroscience: The interplay between Hebbian and homeostatic synaptic plasticity. J Cell Biol 203, 175–186, doi: 10.1083/jcb.201306030 (2013). 24165934 PMC3812972

[b50] Sutton M. A. *et al.* Miniature neurotransmission stabilizes synaptic function via tonic suppression of local dendritic protein synthesis. Cell 125, 785–799, doi: 10.1016/j.cell.2006.03.040 (2006). 16713568

[b51] Hung A. Y. *et al.* Smaller dendritic spines, weaker synaptic transmission, but enhanced spatial learning in mice lacking Shank1. J Neurosci 28, 1697–1708, doi: 10.1523/JNEUROSCI.3032-07.2008 (2008). 18272690 PMC2633411

[b52] Muddashetty R. S., Kelic S., Gross C., Xu M. & Bassell G. J. Dysregulated metabotropic glutamate receptor-dependent translation of AMPA receptor and postsynaptic density-95 mRNAs at synapses in a mouse model of fragile X syndrome. J Neurosci 27, 5338–5348, doi: 10.1523/JNEUROSCI.0937-07.2007 (2007). 17507556 PMC6672337

[b53] Restivo L. *et al.* Enriched environment promotes behavioral and morphological recovery in a mouse model for the fragile X syndrome. Proc Natl Acad Sci USA 102, 11557–11562, doi: 10.1073/pnas.0504984102 (2005). 16076950 PMC1183589

[b54] Todd P. K., Mack K. J. & Malter J. S. The fragile X mental retardation protein is required for type-I metabotropic glutamate receptor-dependent translation of PSD-95. Proc Natl Acad Sci USA 100, 14374–14378, doi: 10.1073/pnas.2336265100 (2003). 14614133 PMC283599

[b55] Zalfa F. *et al.* A new function for the fragile X mental retardation protein in regulation of PSD-95 mRNA stability. Nat Neurosci 10, 578–587, doi: 10.1038/nn1893 (2007). 17417632 PMC2804293

[b56] Feng G. *et al.* Imaging neuronal subsets in transgenic mice expressing multiple spectral variants of GFP. Neuron 28, 41–51 (2000). 11086982 10.1016/s0896-6273(00)00084-2

[b57] Hirasawa T. *et al.* Visualization of microglia in living tissues using Iba1-EGFP transgenic mice. J Neurosci Res 81, 357–362, doi: 10.1002/jnr.20480 (2005). 15948177

[b58] Pardridge W. Peptide drug delivery to the brain. pp. 1–357 (Raven Press, 1991).

[b59] Paxinos G. F. K. The Mouse Brain in Stereotaxic Coordinates. Second edn, (Academic, 2001).

[b60] Schweitz H. *et al.* Calcicludine, a venom peptide of the Kunitz-type protease inhibitor family, is a potent blocker of high-threshold Ca^2+^ channels with a high affinity for L-type channels in cerebellar granule neurons. Proc Natl Acad Sci USA 91, 878–882 (1994). 8302860 10.1073/pnas.91.3.878 PMC521415

[b61] McDonough S. I., Boland L. M., Mintz I. M. & Bean B. P. Interactions among toxins that inhibit N-type and P-type calcium channels. J Gen Physiol 119, 313–328 (2002). 11929883 10.1085/jgp.20028560 PMC2311392

[b62] Newcomb R. *et al.* Selective peptide antagonist of the class E calcium channel from the venom of the tarantula Hysterocrates gigas. Biochemistry 37, 15353–15362, doi: 10.1021/bi981255g (1998). 9799496

