# Supplementary material for: Abnormal intrinsic dynamics of dendritic spines in a fragile X syndrome mouse model in vivo
Source: Sci Rep. 2016 May 25;6:26651. doi: 10.1038/srep26651 (PMC4879559; doi:10.1038/srep26651)
Supplement: Supplementary Information [file srep26651-s1.doc]

Supplementary Information

Abnormal intrinsic dynamics of dendritic spines in a fragile X syndrome mouse model *in vivo*

Akira Nagaoka, Hiroaki Takehara, Akiko Hayashi-Takagi, Jun Noguchi, Kazuhiko Ishii, Fukutoshi Shirai, Sho Yagishita, Takanori Akagi, Takanori Ichiki, and Haruo Kasai

**Supplementary Figure S1**


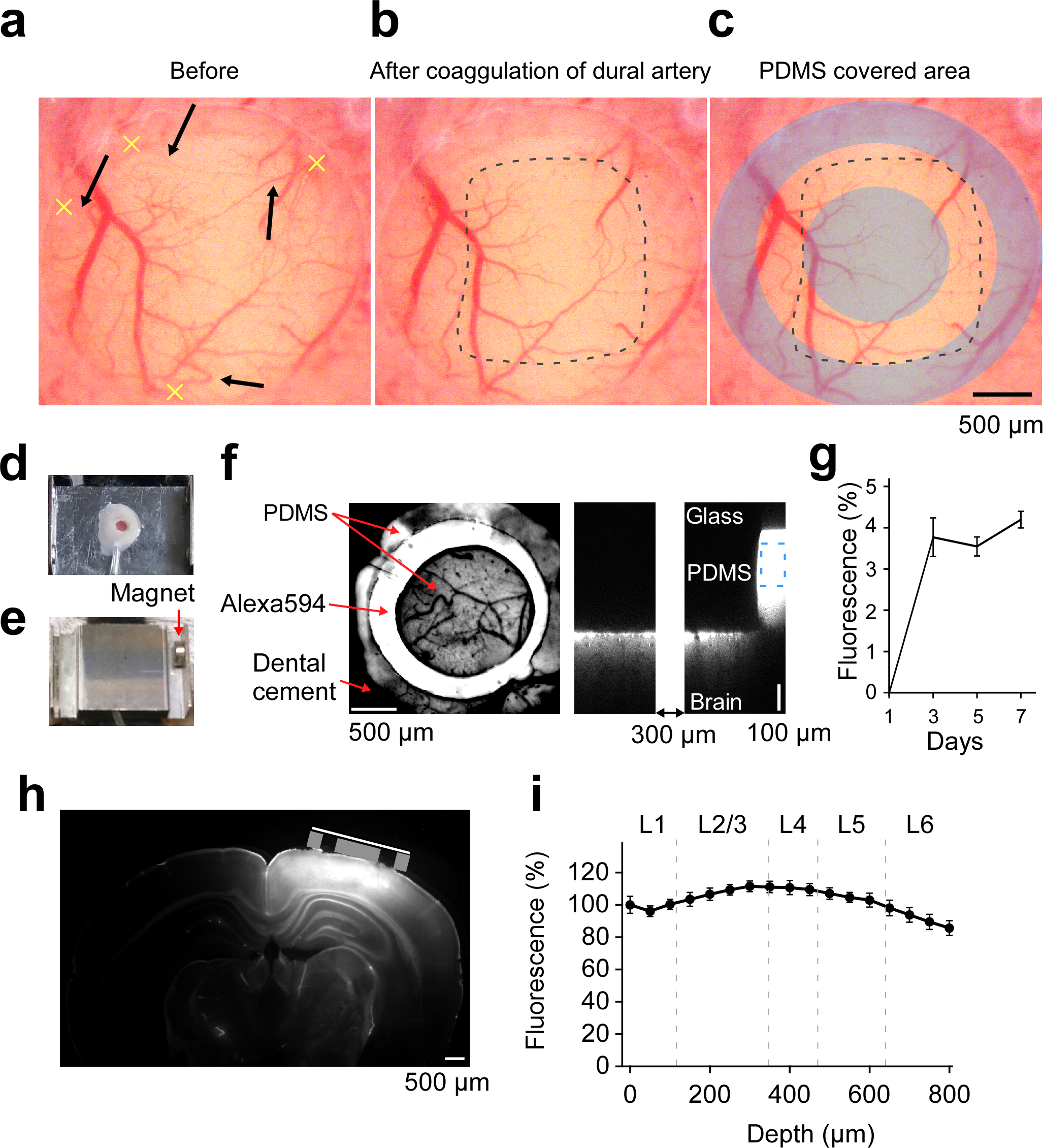


**Supplementary Figure 1.** **Cortical surface infusion with an interface device *in vivo***

(**a**–**c**) Blood vessels in the cranial window prior to (**a**) and after (**b**) coagulation (yellow cross) of blood vessels (arrows) in the region of the dura intended for removal (the dashed square), to prevent bleeding after surgery. Blue-shaded areas in (**c**) indicate the areas covered with poly (dimethylsiloxane) (PDMS). (**d**) The cranial windows during imaging. (**e**) Protection of the cranial window for mice returned to their home cage. (**f**) Fluorescence images of the cortical surface through the interface device. The left panel displays an *xy*-image and the two right panels show *xz*-images crossing the center of the device. (**g**) The time course of Alexa 594 fluorescence intensity within the blue dashed rectangle in (**f)** prior to (day 1) and after (days 3–7) infusion relative to the original solution in the osmotic pump. (**h**) A fluorescence image of a fixed slice from an Alexa 594-infused mouse brain, with the interface device illustrated to indicate where it was placed. (**i**) Fluorescence from a fixed slice of Alexa594-superfused mouse brain indicates that the concentration was not significantly reduced up to 800 μm from the surface. Data are represented as mean ± SEM.

**Supplementary Figure S2**

**
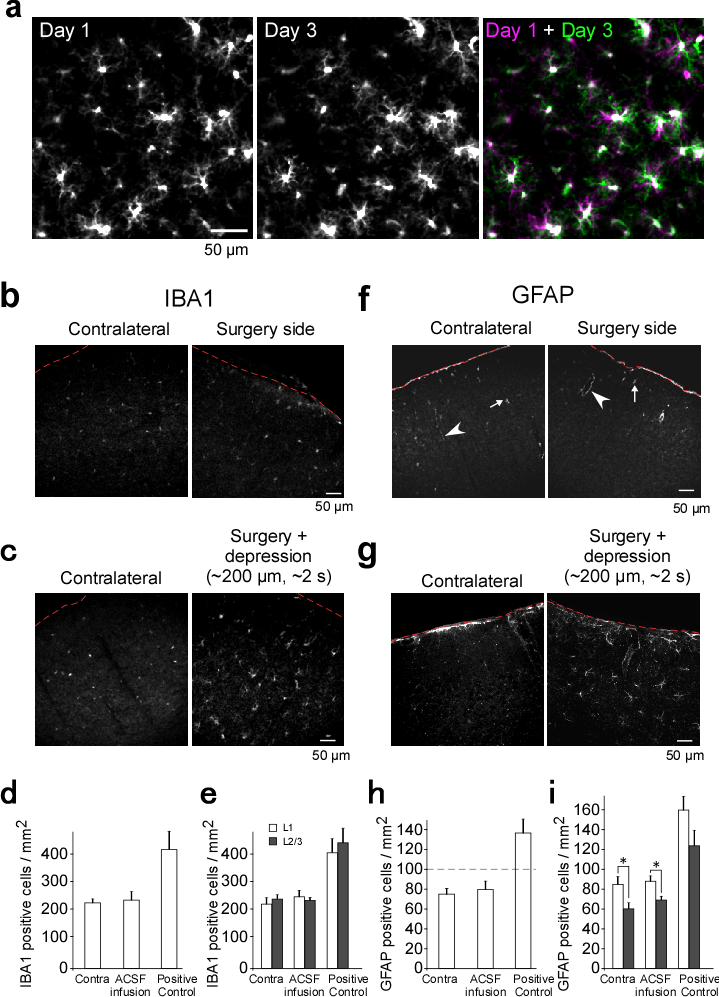
**

**Supplementary Figure 2.** **The absence of microglia and astrocyte activation following interface infusion for 2 days**

(**a**) Microglia demonstrated limited migration in days 1 and 3 following infusion *in vivo*. (**b** and **c**) Anti-IBA1 staining of microglia in artificial cerebrospinal fluid (ACSF) infused mice (**b**) and a positive control (**c**) in the area beneath the device (right) and the contralateral area (left). Red dashed lines indicate the pial surface. (**d**) Average densities of IBA1-positive cells. (**e**) Comparison of the densities of IBA1-positive cells in layers II/III and I. (**f** and **g**) Anti-GFAP staining of astrocytes in ACSF infused mice (**f**) and a positive control (**g**) in the area beneath the device (right) and contralateral area (left). White arrows indicate examples of GFAP-positive astrocytes in **h**, arrowheads indicate GFAP-positive astrocytes surrounding blood vessels. (**h**) Average densities of GFAP-positive cells. (**i**) Comparison of the densities of GFAP-positive cells in layers II/III and I. Data are represented as mean ± SEM.

**Supplementary Figure S3**

**
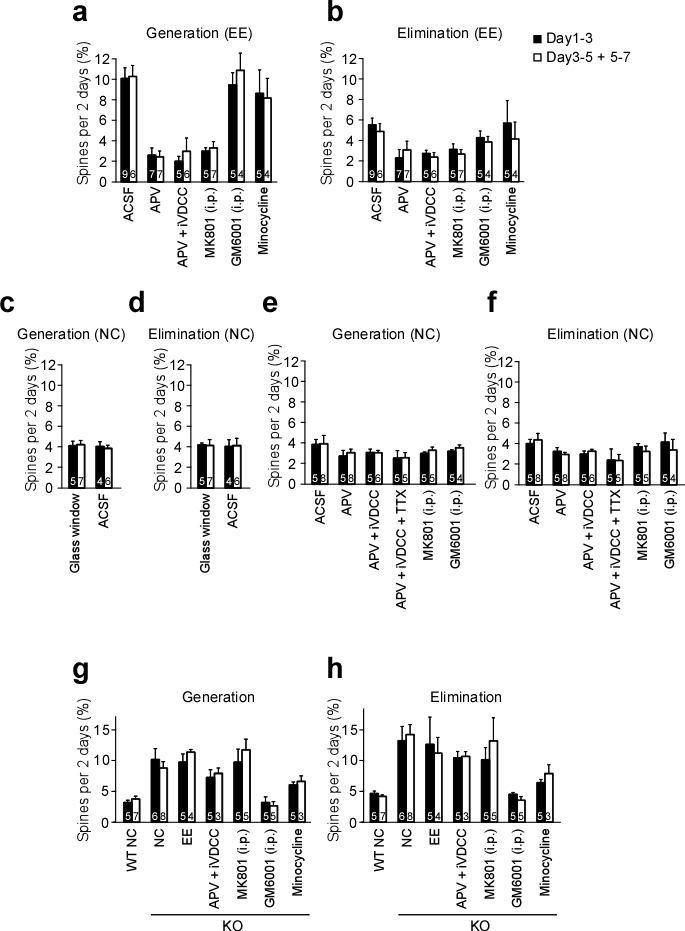
**

**Supplementary Figure 3. Generation and elimination rates in initial and subsequent imaging intervals**

Spine turnover with regard to every 2-day interval in wild-type and knockout (KO) mice during the first imaging interval (days 1–3, filled) and in the subsequent 2-day imaging intervals (open). The experimental conditions are the same as shown in **Fig. 2 and 3**. Bars refer to (**a**) generation and (**b**) elimination of spines in environment enriched (EE) conditions (related to **Fig. 2g** and **h**); (**c**) generation and (**d**) elimination of spines in mice with implanted glass window or microfluidic devices with infusion of artificial cerebrospinal fluid (ACSF) (related to **Fig. 2c** and **d**); (**e**) generation and (**f**) elimination of spines in normal conditions (NC) (related to **Fig. 2k** and **l)**; and (**g**) generation and (**h**) elimination in KO mice (related to **Fig. 3c** and **f)**. The numbers on each bar indicate the number of intervals analyzed. Data are represented as mean ± SEM.

**Supplementary Figure S4**

**
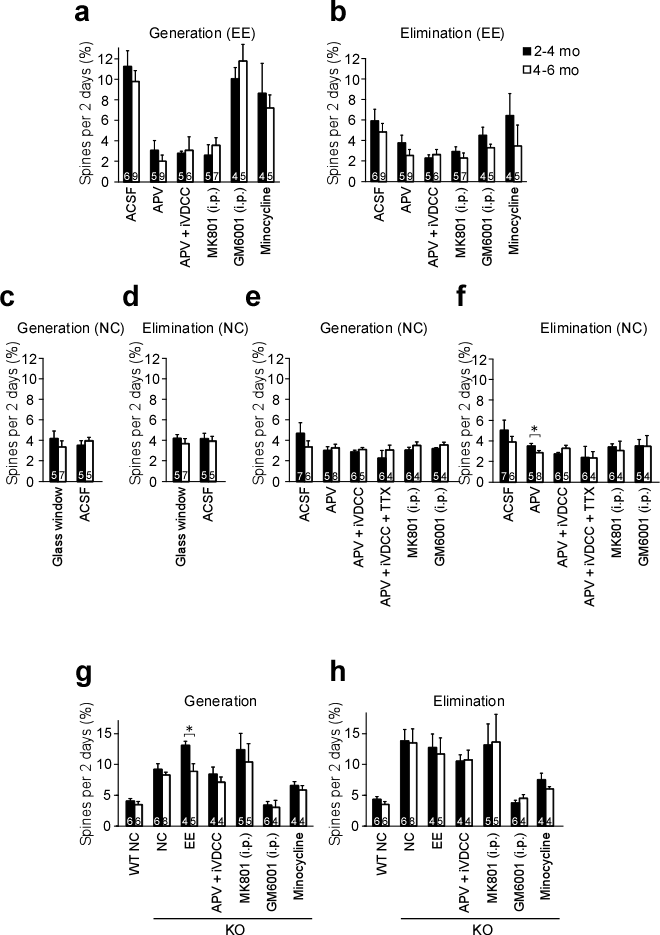
**

**Supplementary Figure 4. Generation and elimination rates in 2–4- and 4–6-month-old mice**

Spine turnover during 2-day intervals in wild-type and knockout (KO) mice aged 2–4 (filled) and 4–6 months (open) were mostly comparable under both conditions, except for the APV group under normal conditions (NC) (**f**, **p* < 0.05) and the KO group under environmentally enriched (EE) conditions (**g**, **p* < 0.05). Experimental conditions are the same as shown in **Fig. 2 and 3**. The numbers on each bar indicate the number of intervals analyzed. Data are represented as mean ± SEM.
